# Paradoxical Embolism due to Persistent Foramen Ovale; a Case Report

**Published:** 2017-01-14

**Authors:** Dormar David Barrios, Jonathan Roncancio, Albert Alejandro Avila, Jaime Andrés Alvarado, Ana Cristina Montenegro

**Affiliations:** 1 Emergency Department, Fundacion Santa Fe De Bogota Hospital, Bogota, Colombia.

**Keywords:** Embolism, Paradoxical, blood coagulation, Anticoagulants, emergency department, venous thromboembolism

## Abstract

The mean percentage of cryptogenic strokes among ischemic strokes is 31%, of which one-third may be associated with patent foramen ovale. The foramen ovale is required for blood flow through the fetal atrial septum. It is formed as of the fourth week of gestation, and this leads to right-left interatrial shunt that allows the passage of oxygenated blood to systemic circulation. In 75% of cases, its closure is complete by 2 years of age, but it may persist in 25% of patients. We present the case of a patient with paradoxical embolism in the lower extremities and ischemic stroke in the clinical context of a patent foramen ovale.

## Introduction

The mean percentage of cryptogenic strokes among ischemic strokes is 31%, of which one-third may be associated with patent foramen ovale(1). The foramen ovale is required for blood flow through the fetal atrial septum. It is formed as of the fourth week of gestation, and this leads to right-left interatrial shunt that allows the passage of oxygenated blood to systemic circulation. In 75% of cases, its closure is complete by 2 years of age, but it may persist in 25% of patients(2). Here a rare presentation of embolism was reported as an excuse to discuss a special aspect of paradoxical thromboembolism.

## Case presentation

A64-year-oldfemale patient was admitted to emergency department due to sudden asthenia, adynamia, vertiginous sensation, and feeling faint. Subsequently, she reports retrosternal chest pain that was associated with dyspnea, dysarthria, bradypsychia, and hypoprosexia. Therefore, the immediate response team goes into action for stroke code activation. Medical history of importance was that she underwent left knee arthroscopy three days before admission. Her vital signs on arrival were as follows: blood pressure, 90/60 mmHg; heart rate 59 beats/minute; respiratory rate, 18 breaths/minute; and axillary temperature, 36.9 °C.

**Figure 1:**
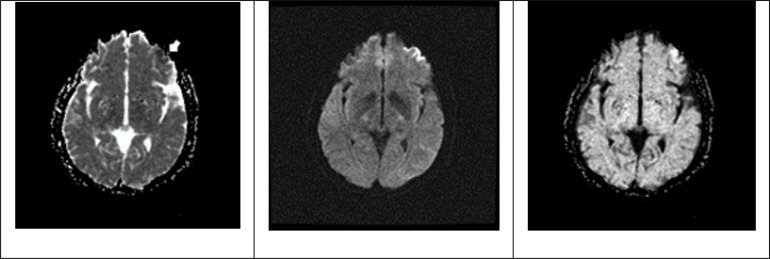
Brain magnetic resonance imaging (MRI) and magnetic resonance angiography (MRA).

From a neurological point of view, she was alert, disoriented in time with fluctuating nomination, repetition alteration, phonetic, semantic paraphasias,and hypoesthesia in right side of body.Brain magnetic resonance imaging (MRI)and magnetic resonance angiography (MRA) shows small acute infarctions at the left frontal cortical, probably due to hypo-perfusion or micro emboli (Figure 1). Afterwards, a transesophageal echocardiogram was carried out, which shows a foramen ovale that allows passage of abundant amount of bubbles to the left atrium, associated with severe tricuspid valve insufficiencyand high probability of pulmonary hypertension (pulmonary artery systolic pressure of 58 mmHg).

**Figure 2:**
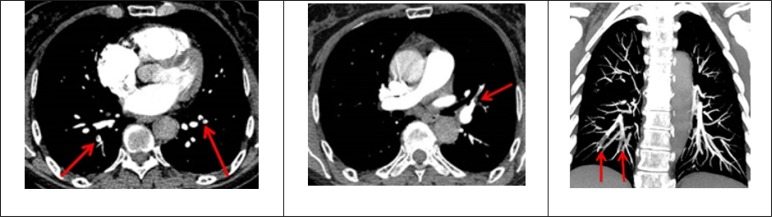
Axial and coronal plan cuts of chest angiogram

Due to the findings from the echocardiogram, a ventricular B-type Natriuretic Peptide (BNP) test was conducted with a result of 132 pg/ml, as well as high sensitivity troponin I assay with a result of 1044 ng/dl (reference value 50 ng/dl). The pretest probability was calculated using the Wells’ scale and a high probability was obtained. Therefore, a computed tomography (CT) angiography was carried out, and it showed evidence of pulmonary thromboembolism which jeopardizes the segmental and sub-segmental branches of the upper lobes, lingula, middle lobe, and both lower lobes (Figure 2).

The risk of paradoxical embolism (RoPE) score was calculated, and it shows an intermediate probability. The pulmonary embolism severity index (PESI) score was determined to be 154 points that corresponds to a class V risk (very high risk). From the above findings, paradoxical embolism has manifested itself due to vascular ischemic attack and pulmonary embolism of intermediate risk as a result of elevated troponin levels and signs of right ventricular dysfunction, in the context of a patent foramen ovale.

Full anticoagulation treatment was started with heparins of low molecular weight, enoxaparin, and the patient is considered not to be a suitable candidate for thrombolysis with instructions to be monitored in an intensive care unit (ICU). 

The patient satisfactorily evolved and in view of clinical improvement, the patient is discharged with the Factor Xa inhibitor (Apixaban) without a loading dose. Treatment was continued for 6 months, in addition to physical rehabilitation and language therapy.

An outpatient control was held a month after the event, and major neurological improvement was evidenced but paraphasia is still present.

## Discussion

Although the majority of people with patent foramen ovale are asymptomatic, this can become a transit channel for venous/arterial emboli, also known as paradoxical embolism. It can lead to a variety of clinical conditions such as stroke with an increase of twice the risk of death and disability in the long term(3).

Paradoxical embolism is defined as the passage of a thrombus from the venous circulatory system to the systemic circulation. For it to occur, specific clinical conditions, such as the following, must be fulfilled(4):

1. In the majority of cases, it is associated with the presence of risk factors that increase the likelihood of occurrence such as hypercoagulable states, recent post-operation states, being bedridden for prolonged periods, and having a history of neoplastic diseases of malignant etiology.
2. An anatomical communication between the venous system and the systemic circulation is required, as well as patent foramen ovale or defects in the septum at the atrial septum level.
3. Lastly, it is conditioned by an increase of the pressure gradient from right to left, as are Valsalva maneuvers.

For a proper definition, paradoxical embolism is the incidental manifestation of a venous thrombosis with a pulmonary embolism associated with a systemic embolism; which clinically presents itself in the vast majority of cases as an ischemic cerebrovascular event that may be acute or transitory.

The patent foramen ovale can be found in about 30% of patients with ischemic cerebrovascular disease as an only finding(1).

Its prevalence appears to decrease with an increase in age, with an incidence of 34% during the first 3 decades of life, 25% between the third and seventh decade, and less than 20% in the octogenarian stage(5).

The risk of paradoxical embolism was revised in the RoPE Study which marks a large collaborative effort to pool research on cryptogenic stroke and patent foramen ovale. Outcome events have been adjudicated so that recurrent stroke can be modeled excluding those with a non-patent foramen ovale related mechanism(6).

The treatment for secondary prevention is unknown. Antiplatelet therapy and oral anticoagulants in single or combined therapy can be used.Warfarin aspirin recurrent stroke study (WARSS)with a population of 2206, was a double-blind randomized multicenter study that compared acetylsalicylic acid with warfarin in the prevention of ischemic cerebrovascular event. Regardless of the presence of patent foramen ovale, there was no significant difference between warfarin and acetylsalicylic acid(7).

Patent foramen ovale in cryptogenic stroke study(PICCS), another randomized multi-centered study evaluated 630 patients to compare warfarin (INR 1.4 - 2.8) and acetylsalicylic acid (325mg/mg) in patients with patent foramen ovale and stroke. It was noted that there is no significant difference between the two treatment strategies. However, the absolute risk for death or ischemic stroke was reduced by approximately half with the use of warfarin compared with acetylsalicylic acid in the subgroup of patent foramen ovale(8).

In both studies, there was more presence of minor bleeding in the vitamin K antagonist group. In our specific case, oral anticoagulants are the first measure given that the need for thrombolysis in patients with acute pulmonary embolism without hypotension nor shock, places it as an intermediate-low risk and would only be implemented in a selected population. However, as of this moment, tracers that help define which patients will benefit from thrombolysis do not exist. 

Individual tracers of right ventricular dysfunction and myocardial injury have an insufficient positive predictive value for complications and decision-making with regard to this therapy(9).

The pulmonary embolism thrombolysis(PEITHO) study determined the effectiveness and safety of early thrombolytic therapy in normotensive patients with right ventricular dysfunction, detected in an echocardiographic scan or through a computerized axial tomography, or evidence of cardiac ischemic injury through proof of positive troponin(10).However, this study showed that the thrombolytic therapy prevented the hemodynamic decompensation in the future, and increases the risk of severe bleeding and hemorrhagic cerebrovascular disease. Which in the clinical context of our patient was highly probable due to the presence of recent ischemic cerebrovascular disease and the prior orthopedic surgery.

With these findings, the next step was to determine the risk stratification in subgroups to know if they benefited from the early recanalization therapy through the administration of fibrinolytic treatment. The study conducted by Bova et al. demonstrated that three stages (I,II,III) were identified through the calculation of a seven-point risk index, which showed rates of complications related with pulmonary embolism after 30 days at 4.2%, 10.8% and 29.2%, respectively(11).

Again, the risk in our patient was raised, calculating a stage III Bova index, which in view of the presented evidence, defined the need of our patient to undergo thrombolytic therapy to prevent complications and mortality in the next 30 days. However, this would be applicable to patients with only thrombus pulmonary embolism and there is no solid evidence in patients with paradoxical embolism and much less in ischemic stroke. Therefore, for future studies, it is necessary to validate this data and issue suggestions with solid medical evidence in this type of patient, as observed in our case report.
